# Experimental Infection of North American Birds with the New York 1999 Strain of West Nile Virus

**DOI:** 10.3201/eid0903.020628

**Published:** 2003-03

**Authors:** Nicholas Komar, Stanley Langevin, Steven Hinten, Nicole Nemeth, Eric Edwards, Danielle Hettler, Brent Davis, Richard Bowen, Michel Bunning

**Affiliations:** * Centers for Disease Control and Prevention, Fort Collins, Colorado, USA; † Colorado State University, Fort Collins, Colorado, USA; ‡ Office of the Surgeon General, United States Air Force, Bolling Air Force Base, Washington, D.C., USA

**Keywords:** West Nile virus, birds, reservoir competence, experimental infection, cloacal swabs, oral swabs, viral persistence, viral shedding, oral infection, research

## Abstract

To evaluate transmission dynamics, we exposed 25 bird species to West Nile virus (WNV) by infectious mosquito bite. We monitored viremia titers, clinical outcome, WNV shedding (cloacal and oral), seroconversion, virus persistence in organs, and susceptibility to oral and contact transmission. Passeriform and charadriiform birds were more reservoir competent (a derivation of viremia data) than other species tested. The five most competent species were passerines: Blue Jay (*Cyanocitta cristata*), Common Grackle (*Quiscalus quiscula*), House Finch (*Carpodacus mexicanus*), American Crow (*Corvus brachyrhynchos*), and House Sparrow (*Passer domesticus*). Death occurred in eight species. Cloacal shedding of WNV was observed in 17 of 24 species, and oral shedding in 12 of 14 species. We observed contact transmission among four species and oral in five species. Persistent WNV infections were found in tissues of 16 surviving birds. Our observations shed light on transmission ecology of WNV and will benefit surveillance and control programs.

*West Nile virus* (WNV) is a mosquito-borne flavivirus (family: *Flaviviridae*) that uses birds as primary vertebrate reservoir hosts (*1*). WNV emerged in North America in New York City in 1999 (*2*,*3*) and has since spread throughout much of the North American continent (*4*). The virus affects the health of the public as well as domestic animals and wildlife. In 1999–2001, WNV was associated with 149 cases of clinical neurologic disease in humans (e.g., encephalitis and meningitis) (*2*,*4*,*5*), 814 cases of equine encephalitis (*4*–*6*), and 11,932 deaths in birds in the United States (*4*,*5*,*7*). Most reported fatal infections in birds occurred in crows. The American Crow (see Table 1 for scientific names of birds) has been proposed as the basis for a national surveillance system for avian deaths attributed to WNV (*7*,*8*). Since 1999, >150 species of dead birds have been reported as WNV positive to the Centers for Disease Control and Prevention (CDC) ArboNET surveillance program (unpub. data). Although the precise cause of death in these birds may not be proven, WNV has been isolated from the carcasses or WNV-specific RNA sequences have been detected. However, not all birds die from infection with the New York 1999 strain of WNV. Many birds sampled in 1999 and 2000 in New York City survived natural WNV infection and developed humoral immunity (*9*,*10*).

**Table 1:** Classification, sample sizes, types of transmission studies and sources for 25 species of birds infected experimentally with West Nile virus

Common name	Latin name	Family	Order	No. used	Transmission trials^a^	Source type
Canada Goose	*Branta canadensis*	Anatidae	Anseriformes	3	M	Wild
Mallard	*Anas platyrhynchos*	Anatidae	Anseriformes	3	M, C	Commercial
American Kestrel	*Falco sparverius*	Falconidae	Falconiformes	5	M, O	Rehabilitator
Northern Bobwhite	*Colinus virginianus*	Odontophoridae	Galliformes	6	M, C, O	Commercial
Japanese Quail	*Coturnix japonicus*	Odontophoridae	Galliformes	6	M, C, O	Commercial
Ring-necked Pheasant	*Phasianus colchicus*	Phasianidae	Galliformes	3	M	Commercial
American Coot	*Fulica americana*	Rallidae	Gruiformes	2	M, C	Wild
Killdeer	*Charadrius vociferus*	Charadriidae	Charadriiformes	2	M	Wild
Ring-billed Gull	*Larus delawarensis*	Laridae	Charadriiformes	7	M, C	Wild
Mourning Dove	*Zenaida macroura*	Columbidae	Columbiformes	6	M, C, O	Wild
Rock Dove	*Columba livia*	Columbidae	Columbiformes	12	M, C	Commercial
Monk Parakeet	*Myiopsitta monachus*	Psittacidae	Psittaciformes	6	M, C, O	Commercial
Budgerigar	*Melopsittacus undulatus*	Psittacidae	Psittaciformes	6	M, C, O	Commercial
Great Horned Owl	*Bubo virginianus*	Strigidae	Strigiformes	2	M, O	Rehabilitator
Northern Flicker	*Colaptes auratus*	Picidae	Piciformes	5	M, O	Wild
Blue Jay	*Cyanocitta cristata*	Corvidae	Passeriformes	6	M, C	Wild
Black-billed Magpie	*Pica hudsonia*	Corvidae	Passeriformes	8	M, C, O	Wild
American Crow	*Corvus brachyrhynchos*	Corvidae	Passeriformes	22	M, C, O	Wild
Fish Crow	*Corvus ossifragus*	Corvidae	Passeriformes	20	M, C, O	Wild
American Robin	*Turdus migratorius*	Turdidae	Passeriformes	6	M, C, O	Wild
European Starling	*Sturnus vulgaris*	Sturnidae	Passeriformes	8	M, C	Wild
Red-winged Blackbird	*Agelaius phoeniceus*	Icteridae	Passeriformes	4	M	Wild
Common Grackle	*Quiscalus quiscula*	Icteridae	Passeriformes	12	M, C, O	Wild
House Finch	*Carpodacus mexicanus*	Fringillidae	Passeriformes	3	M, C, O	Wild
House Sparrow	*Passer domesticus*	Passeridae	Passeriformes	15	M, C, O	Wild

^a^M, mosquito-exposed; C, contact-exposed; O, orally exposed.

Although crows are commonly reported as infected with WNV (*11*), the identity of the avian reservoirs for WNV remains unknown. Surveillance data on avian deaths and seroprevalence studies suggest hypotheses about reservoir host species but do not indicate the competence of a particular species to infect a culicine vector. Furthermore, birds may be involved in transmission by means other than mosquito bites, yet little is known about contact or oral transmission among birds.

To better understand the role of birds in WNV transmission, we exposed 25 species of birds, representing a wide range of avian orders and families, to infectious mosquito bites. We then monitored viremia titers, clinical outcomes, viral shedding in cloacal and oral cavities, persistence of viral infections in organs, and development of neutralizing antibodies. The viremia data generated were used to quantitate reservoir competence. We also evaluated susceptibility to oral and direct contact transmission when possible.

## Methods

### Source of Birds

Birds were obtained commercially when possible or as nonreleasable injured birds (raptors only), or from the wild (Table 1). Only seronegative birds were used. Information on the plaque-reduction neutralization assay used is available in Appendix A.

### Source and Infection of Mosquitoes

We used colonized mosquitoes (*Culex tritaeniorhynchus*) originally obtained from Taiwan in 1997. Adult female mosquitoes (<10 days old), used for infecting birds, were inactivated by chilling at approximately 4°C and inoculated intrathoracically with 1 µL of an aqueous solution containing 10^7^ PFU WNV (NY99-6480) per 1 mL. Mosquitoes were then incubated at 16:8 h light:dark, 28°C, 80% relative humidity for 6–10 days before they were exposed to birds. Successful infection of mosquitoes was confirmed by plaque assay of whole mosquito homogenates (after incubation).

### Source of Virus

Two isolates of WNV (New York 1999) were used. The NY99-6480 strain was isolated from mosquitoes (*C. pipiens*) and passed once in Vero cell culture. The NY99-4132 strain was isolated from brain of an American Crow and passed one to three times in Vero cell culture. The TBH-28 strain of *St. Louis encephalitis virus* (SLEV; family: *Flaviviridae*) was obtained from the CDC reference collection.

### Experimental Infection

We exposed birds to WNV-infectious mosquito bites by holding their exposed skin (usually of the breast) against a screened carton containing 5–15 mosquitoes. Birds were considered sufficiently exposed when one mosquito had engorged to repletion. In the few cases when birds were probed extensively by mosquitoes but no visible blood was imbibed, we considered them infected if viremia developed. When possible, at least one uninfected conspecific bird (contact-exposed group) was placed in a cage with a mosquito-exposed bird as a control for direct transmission (in the absence of mosquito-borne transmission). Some birds (orally exposed group) were exposed to per os infections by using a variety of techniques; our objective was to show that per os transmission is possible. Techniques used included placing 200 uL water (containing a suspension of WNV [NY99-4132]) in the back of the oral cavity to stimulate the swallow reflex; placing a dead infected mosquito (containing approximately 10^7^ PFU) in the bird’s oral cavity and stimulating the swallow reflex with 200 uL of water; and placing a dead infected adult House Mouse (*Mus musculus*) or House Sparrow (euthanized 3–5 days after subcutaneous injection of 2,000–8,000 PFU) in the cage. Viral loads in the mice and House Sparrows were inferred from infected cohorts and estimated at >10^5^ PFU per animal. Information about methods for venipuncture is available in Appendix B.

### Collection of Oral and Cloacal Samples

For some birds, daily cloacal or nasopharyngeal (oral) swabs were collected concurrently with blood samples during the first 7 days postinoculation (dpi). Cotton- or dacron-tipped applicators were used, and contaminated swabs were dipped in cryovials containing 0.5-mL BA1 to transfer any virus to the cryovial. These cryovials were placed immediately on wet ice (temporarily) and stored at –70°C for subsequent titration by Vero plaque assay (described in Appendix C).

### Illnesses, Deaths, and Euthanasia

Exposed birds were observed twice a day for signs of severe illness, such as neurologic irregularities and recumbency. Birds unable to ambulate or consume food and water were euthanized by CO_2_ asphyxiation or intravenous inoculation of sodium pentabarbitol at a dose of approximately 80 mg/kg. We recorded fatal cases to determine estimates of mortality rates for each species.

### Necropsy

At the close of each infection study (in most cases 14 dpi), surviving birds were euthanized. Necropsies were performed immediately or after carcasses were stored at –70°C. Eleven organs were sampled for each bird by removing approximately 0.5 cm^3^ to a sterile TenBroeck tissue homogenizer containing alundum grinding crystals and 0.2 mL BA1, 20% fetal calf serum. After grinding, 1.8 mL BA1, 20% fetal calf serum was added to each homogenate, and then each homogenate was transferred to 1.7-mL Eppendorf tubes for clarification by centrifugation at 7,500 rpm for 3 min. Supernatants were transferred to cryovials for storage at –70°C until titrated by plaque assay.

### Calculation of Reservoir Competence Values

An index of reservoir competence (*C_i_*) was derived as the product of three factors: susceptibility (*s*), the proportion of birds that become infected as a result of exposure; mean daily infectiousness (*i*), the proportion of vectors that become infected per day; and duration (*d*) of infectiousness, the number of days that a bird maintains an infectious viremia (*12*). This simple equation can be expressed as C_i_ = s _*_ i _*_ d. Thus, the competence index indicates the relative number of infectious vectors that derive from a particular bird species and is calculated as a function of the viremia that develops after mosquito-borne infection. To produce these data, we used a threshold level of infectious viremia of 10^5.0^ PFU/mL serum and estimated infectiousness of each bird’s viremia levels from a standard curve for infection of *C. pipiens* as a function of viremic titer derived from Turell et al. (*13*) (Appendix D).

## Results

### Viremia Profiles

**Figure 1:**
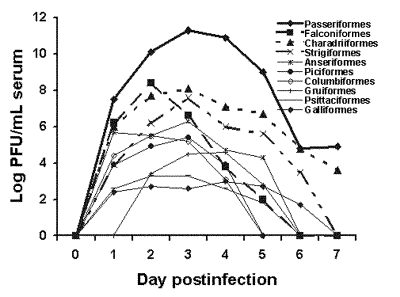
Comparative West Nile virus viremia profiles for 10 orders of birds.

We determined WNV viremia profiles for 25 species of birds representing 17 families and 10 orders (Figure 1). Four (a Budgerigar, a Monk Parakeet, and two Japanese Quail) of 87 birds did not develop a detectable viremia (threshold of detection 50 PFU/mL serum) (Table 2). Four birds sustained detectable viremias of 7 days (a Ring-billed Gull, a House Finch, and two Fish Crows). Fish Crows were bled daily after 7 dpi for an additional 4 days to investigate whether viremias may endure >7 days (Table 3). They did not, although one moribund Fish Crow became viremic at 11 dpi, shortly before dying. Generally, viremias averaged greater in magnitude and duration in passerine and charadriiform birds than in other orders. Psittacine and gallinaceous birds had the lowest titered and shortest duration viremias.

**Table 2:** Mean West Nile virus viremias (shown as log_10_ PFU/mL serum, with ranges) for each of 7 days postinoculation by mosquito bite, and mean duration of detectable viremia (days, with ranges)^a,b^

Species	Day postinoculation								Duration of viremia
	n	1	2	3	4	5	6	7	
Canada Goose	3	2.8 (<1.7–3.0)	5.3 (3.2–5.8)	4.5 (3.5–4.8)	3.4 (<1.7–3.8)	1.9 (<1.7–2.0)	<1.7	<1.7	4.0 (3–5)
Mallard	2	6.1 (<1.7–6.4)	5.7 (5.5–5.9)	6.7 (3.4–7.0)	5.1 (1.7–5.4)	4.7 (<1.7–5.0)	<1.7	<1.7	4.0 (4–4)
American Kestrel	2	6.2 (5.5–6.4)	8.4 (5.8–8.7)	6.6 (6.1–6.8)	3.8 (3.6–4.0)	2 (<1.7–2.3)	<1.7	<1.7	4.5 (4–5)
Northern Bobwhite	3	2.8 (1.7–3.1)	2.9 (2.3–3.3)	2.0 (1.7–2.2)	1.9 (<1.7–2.4)	1.2 (<1.7–1.7)	<1.7	<1.7	4.0 (3–5)
Japanese Quail	3	<1.7	<1.7	2.8 (<1.7–3.3)	3.4 (<1.7–3.9)	3.1 (<1.7–3.6)	2.2 (<1.7-2.7)	<1.7	1.3 (0–4)
Ring-necked Pheasant	3	2.1 (<1.7–2.3)	2.8 (<1.7–3.2)	2.6 (1.7–3.0)	1.8 (1.7–2.0)	1.2 (<1.7–1.7)	<1.7	<1.7	3.7 (3–4)
American Coot	1	<1.7	3.4	4.5	4.6	2.8	<1.7	<1.7	4.0
Killdeer	2	6.2 (5.9–6.4)	7.5 (6.5–7.8)	8.1 (4.3–8.4)	4.9 (2.1–5.2)	2.6 (<1.7–2.9)	<1.7	<1.7	4.5 (4–5)
Ring-billed Gull	2	5.4 (5.4–5.4)	7.8 (6.5–8.1)	8.0 (5.4–8.3)	7.4 (2.9–7.7)	7.2	5.3	4.1	5.5 (4–7)
Mourning Dove	3	4.8 (3.0–5.3)	5.9 (3.9–6.3)	5.6 (3.4–5.9)	3.3 (<1.7–3.6)	<1.7	<1.7	<1.7	3.7 (3–4)
Rock Dove	6	3.5 (<1.7–4.0)	4.3 (3.5–4.8)	4.2 (3.5–4.5)	2.9 (<1.7–3.7)	NT	NT	NT	3.2 (3–4)
Monk Parakeet	3	2.8 (<1.7–3.0)	3.6 (<1.7–4.0)	3.5 (<1.7–3.9)	1.7 (<1.7–2.0)	<1.7	<1.7	<1.7	2.7 (0–4)
Budgerigar	3	2.3 (<1.7–2.8)	1.9 (<1.7–2.4)	2.8 (<1.7–3.3)	2.9 (<1.7–3.4)	2.1 (<1.7–2.6)	<1.7	<1.7	1.7 (0–4)
Great Horned Owl	1	3.9	6.2	7.6	6.0	5.6	3.5	<1.7	6.0
Northern Flicker	1	3.9	4.9	5.4	3.9	<1.7	<1.7	<1.7	4.0
Blue Jay	4	8.5 (5.6–8.8)	11.1 (7.8–11.6)	12.1 (7.5–12.6)	10.5 (5.0–11.0)	2.2	<1.7	<1.7	4.0 (3–5)
Black-billed Magpie	3	5.3 (3.7–5.7)	8.3 (7.7–8.6)	8.8 (8.4–9.1)	4.9 (4.8–5.0)	4.0 (3.9–4.0)	–	–	5.0 (5–5)
American Crow	8	5.8 (<1.7–6.6)	8.7 (<1.7–9.6)	9.9 (6.7–10.6)	10.2 (9.2–10.8)	10.0 (8.2–10.4)	–	–	3.8 (3–5)
Fish Crow	9	5.4 (3.0–6.2)	6.8 (5.6–7.4)	7.8 (5.5–8.7)	8.9 (<1.7–9.9)	8.5 (<1.7–9.5)	4.0 (0-4.9)	1.3 (<1.7-2.0)	5.0 (4–7)
American Robin	2	5.8 (5.6–5.9)	8.9 (7.8–9.2)	7.3 (6.8–7.5)	4.6 (3.7–4.9)	2.0 (<1.7–2.3)	<1.7	<1.7	4.5 (4–5)
European Starling	6	5.3 (3.5–6.0)	6.1 (5.3–6.5)	4.9 (2.0–5.4)	2.3 (<1.7–3.1)	<1.7	<1.7	<1.7	3.2 (3–4)
Red-winged Blackbird	3	5.9 (5.5–6.1)	8.6 (7.5–9.0)	6.0 (5.5–6.3)	<1.7	<1.7	<1.7	<1.7	3.0 (3–3)
Common Grackle	6	6.1 (3.3–6.8)	10.2 (5.4–11.0)	11.8 (4.7–12.5)	11.8 (<1.7–12.5)	<1.7	<1.7	<1.7	3.3 (3–4)
House Finch	2	5.4 (2.3–5.7)	5.8 (5.6–6.0)	8.8 (8.6–8.9)	6.6 (6.0–6.8)	6.0 (5.9–6.1)	6.2	6.3	6.0 (5–7)
House Sparrow	6	7.8 (3.9–8.6)	9.8 (7.6–10.5)	10.3 (4.8–11.0)	10.3 (2.4–11.0)	8.4 (<1.7–9.0)	1.8 (<1.7-2.1)	<1.7	4.5 (2–6)

^a^NT, not tested; –, no birds survived to be sampled. ^b^Log_10_-transformed mean peak viremias ranged from 3.0 for Ring-necked Pheasants (range 2.0–3.2) and Budgerigars (range <1.7–3.4) to 12.1 for Blue Jays (range 7.8–12.6). Mean duration (in days) of detectable viremias ranged from 1.3 in Japanese Quail to 6.0 in House Finches and a Great Horned Owl.

**Table 3:** Daily viremia determinations for nine Fish Crows infected with West Nile virus by mosquito bite^a^

Bird no.	Day postinoculation										
	1	2	3	4	5	6	7	8	9	10	11
015	3.6	5.6	5.7	5.7	<1.7	<1.7	<1.7	<1.7	<1.7	<1.7	<1.7
016	5.4	6.5	5.9	3.4	<1.7	<1.7	<1.7	<1.7	<1.7^b^		
036	4.6	5.6	5.6	3.9	4.7	4.9	2.0	<1.7	<1.7	dead	
038	4.3	5.9	5.5	4.6	3.0	2.8	1.7	<1.7	<1.7	<1.7	3.3^c^
049	4.7	7.0	6.9	4.7	2.4	<1.7	<1.7	<1.7	<1.7	<1.7	<1.7
050	3.0	7.4	8.7	9.9	9.5	dead					
058	5.0	6.7	6.2	5.3	3.3	2.3	<1.7	<1.7	dead		
403	6.2	6.8	5.7	3.0	<1.7	<1.7	<1.7	NT	NT	NT	NT
404	3.1	5.7	7.0	<1.7	3.3	<1.7	<1.7	NT	NT	NT	NT

^a^Values shown are log_10_ transformed and represent the number of PFU/mL serum. ^b^Moribund/euthanized. ^c^Dead at 12 days postinoculation.

### Illness and Death

Of the 87 mosquito-exposed birds, we observed obvious signs of illness in 28 birds, including members of certain passerine species (in particular, the corvids) and the Ring-billed Gull. Signs of illness included generalized lethargy, ruffled feathers, unusual posture (Blue Jay), inability to hold head upright (Ring-billed Gull), and ataxia (Ring-billed Gull). In most cases, clinical signs were followed by death within 24 h. Moribund birds were euthanized, although ill birds were rarely found moribund because death occurred rapidly. External hemorrhage, either from the mouth or from the cloaca, was noted in a small number of American Crows that died. Although our sample sizes and controls were insufficient to generate accurate estimates of mortality rates, our observations can be used to generate preliminary estimates (Table 4).

**Table 4:** Illness observed in eight species of birds exposed to West Nile virus (WNV) by mosquito bite^a^

Species	No. exposed	No. unexposed^b^	No. fatal infections (% exposed)	Days postinoculation that death occurred	Mean no. days to death (range)
Ring-billed gull	2	0	2 (100)	5, 13^c^	9.0 (5–13)
Blue Jay	4	0	3 (75)	4, 5, 5	4.7 (4–5)
Black-billed Magpie	3	0	3 (100)	6, 6, 6	6.0 (6–6)
American Crow	8	8	8 (100)	4, 4, 5, 5, 5, 6, 6, 6	5.1 (4–6)
Fish Crow	9	0	5 (55)	6, 9, 10, 10,^c^ 13	9.6 (6–13)
Common Grackle	6	6	2 (33)	4, 5	4.5 (4–5)
House Finch	2	3	2 (100)	6, 8	7.0 (6–8)
House Sparrow	6	5	3 (50)	3, 5, 6	4.7 (3–6)

^a^Preliminary mortality rates were highest in the Passerines, especially the corvids. No signs of clinical illness were observed among species of the following orders: Anseriformes, Ciconiiformes, Galliformes, Gruiformes, Columbiformes, Psittaciformes, Strigiformes, and Piciformes. No obvious differences in mortality rates were observed among birds exposed to WNV by means other than mosquito bite (orally exposed and contact-exposed groups; data not shown). ^b^Unexposed controls were blood sampled daily for the same period as the exposed birds, with no resulting illness. ^c^Euthanized.

### Oral Transmission

**Figure 2:**
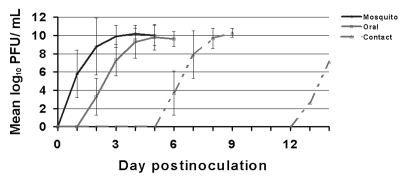
West Nile virus viremia profiles in American Crows that were mosquito-exposed (n=8), orally exposed by ingestion of sparrow carcasses (n=5), or contact-exposed (n=4). A fifth contact-exposed crow developed an ephemeral low-titered viremia (10^2.2^/mL serum) and was treated as an outlier in this analysis. Error bars show standard deviation of log_10_-transformed viremia titers.

We evaluated oral susceptibility to WNV infection for 15 species of birds representing 11 families and seven orders (Table 1). We confirmed susceptibility to orally acquired WNV infection in Great Horned Owl, American Crow, Common Grackle, House Finch, and House Sparrow. The owl that ingested infected mice developed viremia and seroconverted. American Crows also became infected after consuming a WNV-infected House Sparrow carcass (83% susceptibility, n=6); three Black-billed Magpies and a Fish Crow did not become infected after consuming infected House Sparrows or infected mice. American Crows and House Sparrows became infected after ingesting an aqueous solution containing 10^7.4^ PFU (100% susceptibility; n=6 and n=3, respectively). Grackles became infected after ingesting an aqueous solution containing1,000 PFU (100% susceptibility; n=4) but were resistant to a dose of 100 PFU (n=2). One of two House Finches that ate an infected mosquito, representing a dose of about 10^7^ PFU, became viremic. Three each of Mourning Doves and Budgerigars did not become infected after ingesting an infected mosquito; three each of Japanese Quail and Monk Parakeet and two Bobwhite, did not become infected after ingesting an aqueous suspension containing about 3,400 PFU. Viremias generated from oral infection were similar to those from mosquito bite–derived infection, although the onset of detectable viremia was consistently delayed by at least a day (Figure 2), except for the one House Finch and the Great Horned Owl. Viremia profiles of these birds were similar to their mosquito-exposed counterparts, with no delay in the onset of viremia.

### Contact Transmission

We monitored for direct transmission between mosquito-exposed birds and their cage mates among 18 species of bird representing 12 families and seven orders (Table 1). Transmission to cage mates was detected only in Ring-billed Gulls, Blue Jays, Black-billed Magpies, and American Crows (Table 5). The viremia profile of contact-exposed American Crows was similar to that of mosquito-exposed and orally exposed crows (Figure 2). In contact-exposed American Crows and Black-billed Magpies, onset of viremia occurred subsequent to death of their mosquito-exposed cage mates, suggesting that infection occurred near the time of death of the mosquito-exposed birds. The two contact-exposed Blue Jays both became infected while their mosquito-exposed cage mates were still viremic and apparently healthy. The one contact-exposed Ring-billed Gull that became infected did not develop viremia within 7 days of the inoculation of its two mosquito-exposed companions but was viremic at 14 dpi.

**Table 5:** West Nile virus cage mate transmission trials^a^

Species	No. of cages	No. of mosquito- exposed birds	No. of contact exposed cage mates	No. of transmissions (individuals)	No. of transmissions (cages)	Cage transmission rate
Mallard	1	2	1	0	0	0
Northern Bobwhite	1	3	3	0	0	0
Japanese Quail	1	3	3	0	0	0
American Coot	1	1	1	0	0	0
Ring-billed Gull	1	2	1	1	1	1.0
Mourning Dove	3	3	3	0	0	0
Rock Dove	6	6	6	0	0	0
Monk Parakeet	3	3	3	0	0	0
Budgerigar	3	3	3	0	0	0
Blue Jay	2	2	2	2	2	1.0
Black-billed Magpie	3	3	3	2	2	0.7
American Crow	4	8	5	5	4	1.0
Fish Crow	4	8	9	0	0	0
American Robin	1	2	1	0	0	0
European Starling	2	6	2	0	0	0
Common Grackle	6	6	6	0	0	0
House Finch	1	2	3	0	0	0
House Sparrow	2	6	5	0	0	0

^a^Uninfected birds (contact-exposed group) were placed within cages containing birds (of the same species) that were infected by mosquito bite (mosquito-exposed group). Transmission to uninfected cage mates was determined by development of viremia or seroconversion.

### Development of Neutralizing Antibodies

**Figure 3:**
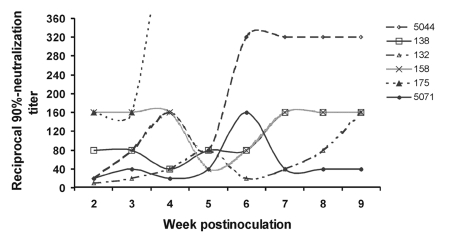
West Nile virus-neutralizing antibody response of six mosquito-exposed Rock Doves (pigeons). Rock Dove 175 reached a titer of 1:640 at 4 weeks postinoculation and then died of other causes.

Most mosquito-exposed birds that survived WNV infection were euthanized at 14 dpi (House Finches were held until 21 dpi, and Rock Doves were held for 64 dpi). We evaluated final serum samples for neutralizing antibodies. Only two birds, both Budgerigars, did not produce at least 70% neutralization activity in the final serum sample (tested at a 1:10 dilution). One of these also did not develop detectable viremia. The other had a detectable viremia only at 24 h postinoculation (log_10_ titer 2.8 PFU/mL serum), which may have represented residual virus from the injection rather than viral multiplication. The neutralizing antibody response of Rock Doves was tracked weekly for 9 weeks postinoculation (Figure 3). Between weeks 2–9 postinoculation, reciprocal 90%-neutralization titers ranged from 10 to 640 and tended to rise early, then fall, and then rise again between weeks 3–7 postinoculation.

### Viral Shedding

Cloacal and in some cases oral (nasopharyngeal) swabs were collected from 24 species of birds that were exposed to mosquitoes for subsequent virus isolation attempts. By swabbing the cloaca, we documented that most birds shed WNV in feces (17 [71%] of 24 species; 46 [59%] of 78 individual birds). Some passerine birds shed large quantities of WNV through the cloaca (Table 6). Cloacal shedding was generally first detected after several days of viremia and persisted longest in Fish Crows (>9 days), with peak cloacal swab titers occurring at 4–5 dpi. Although the highest cloacal swab titers were detected in American Crows and Blue Jays, these did not persist beyond 4 days because the birds died. By swabbing the oral cavity, we documented that most birds shed WNV in oral exudates (11 [85%] of 13 species; 29 [69%] of 42 individuals birds); the highest titers were observed in Great Horned Owl, American Crow, and American Kestrel (Table 7). Shedding per os persisted longest in the American Kestrel (up to 10 days). Shedding (either per cloaca or per os) was observed in representatives of 8 of the 10 orders, with the exceptions of Psittaciformes (n=6 individual birds) and Piciformes species (n=1). Although environmental sampling was not undertaken rigorously, we did detect infectious WNV in a water dish that had been contaminated with Blue Jay feces and from bloody oral effusion collected underneath a dead American Crow.

**Table 6:** West Nile virus shedding in living birds, as determined by daily cloacal swabbing of four species of birds exposed by mosquito bite^a,b^

Species	n	Day postinoculation							No. birds shedding (%)
		1	2	3	4	5	6	7	
Canada Goose	3	<0.2	<0.2	<0.2	<0.2	2.3 (<0.7–2.7)	0.6 (<0.7–1.0)	2.1 (<0.7–2.6)	2 (67)
Mallard	2	<0.4	<0.4	<0.4	<0.4	<0.4	<0.4	<0.4	0
American Kestrel^c^	2	1.6 (1.6–1.6)	2.8 (2.0–3.1)	4.2 (1.9–4.5)	4.9 (4.0–5.2)	4.2 (3.6–4.4)	2.3 (2.2–2.4)	3.1 (<0.4–3.4)	2 (100)
Northern Bobwhite	3	<–0.2	<–0.2	0.2 (<0.4–0.7)	<–0.2	<–0.2	<–0.2	<–0.2	1 (33)
Japanese Quail	3	<–0.2	<–0.2	<–0.2	<–0.2	<–0.2	<–0.2	<–0.2	0
Ring-necked Pheasant	3	<0.2	<0.2	<0.2	<0.2	<0.2	<0.2	<0.2	0
American Coot	1	<0.7	<0.7	<0.7	<0.7	2.7	<0.7	NT	1 (100)
Killdeer	2	<0.1	0.5 (<0.4–0.8)	<0.1	1.5 (<0.4–1.8)	<0.1	<0.1	<0.1	1 (50)
Ring-billed Gull	2	<0.1	<0.1	2.4 (0.4–2.7)	2.3 (2.2–2.5)	<0.4	1.8	<0.4	2 (100)
Mourning Dove	3	<–0.2	1.1 (0.8–1.5)	<–0.2	<–0.2	<–0.2	<–0.2	<–0.2	2 (67)
Rock Dove	6	<–0.5	<–0.5	<–0.5	<–0.5	1.2 (<0.4–1.7)	NT	NT	4 (67)
Monk Parakeet	3	<–0.2	<–0.2	<–0.2	<–0.2	<–0.2	<–0.2	<–0.2	0
Budgerigar	3	<–0.2	<–0.2	<–0.2	<–0.2	<–0.2	<–0.2	<–0.2	0
Great Horned Owl^c^	1	<0.4	2.3	2.1	3.1	2.1	3.3	2.0^c^	1 (100)
Northern Flicker	1	<0.4	<0.4	<0.4	<0.4	<0.4	<0.4	<0.4	0
Blue Jay	4	<–0.3	3.0 (<0.4–3.6)	4.3 (2.1–4.8)	6.0 (2.0–6.4)	2.0	1.0	<0.4	4 (100)
Black-billed Magpie	3	<–0.2	1.8 (<0.4–2.3)	2.4 (2.1–2.5)	1.9 (<0.4–2.3)	2.9 (<0.4–3.4)	–	–	3 (100)
American Crow	6	0.8 (<0.4–1.5)	3.3 (<0.4–4.0)	5.2 (2.7–5.8)	5.0 (3.8–5.5)	5.7	–	–	6 (100)
Fish Crow^d^	8	–0.2 (<0.4–0.7)	1.5 (<0.4–2.1)	3.0 (1.0–3.8)	2.5 (<0.4–3.0)	3.3 (<0.4–4.1)	3.9 (<0.4–4.8)	3.6 (<0.4–4.4)	8 (100)
American Robin	2	<0.4	0.8 (<0.7–1.1)	2.2 (<0.7–2.5)	<0.4	<0.4	<0.4	<0.4	1 (50)
European Starling	6	<–0.5	<-0.5	-0.5 (<0.4–0.4)	<–0.5	–0.5 (<0.4–0.4)	0.8 (<0.4–1.5)	<–0.5	3 (50)
Red-winged blackbird	3	–0.2	0.4 (<–0.4–0.8)	<–0.2	–0.2 (<0.4–0.7)	<-0.2	<–0.2	<–0.2	1 (33)
Common Grackle	6	<–0.5	>2.0 (<0.4–>2.7)	4.5 (0.7–5.3)	5.6 (<0.4–6.4)	5.2 (<0.4–5.9)	0.8 (<0.4–1.4)	0.1 (<0.4–0.7)	6 (100)
House Finch	2	<0.4	<0.4	<0.4	<0.4	<0.4	<0.7	<0.7	0
House Sparrow^e^	6	NT	<–0.5	NT	>1.9 (<0.4–>2.7)	1.3 (<0.4–2.1)	NT	NT	2 (33)

^a^Presented as mean log_10_ PFU/swab, with ranges. ^b^NT, not tested; –, no birds survived the infection to be sampled. ^c^American Kestrels and the Great Horned Owl were tested at 9–11 days postinoculation, with no detectable virus in swabs. ^d^Surviving Fish Crows were tested at 8 and 9 days postinoculation with the following results for day 8 and day 9, respectively: 1.2 (<0.4–1.8); 1.5 (<0.4–2.1). ^e^These house sparrows were inoculated by needle rather than by mosquito bite.

**Table 7:** West Nile virus shedding in living birds, as determined by plaque assay of oral swabs collected daily from 14 species of birds exposed by mosquito bite^a,b^

Species	n	Day postinoculation							No. birds shedding (%)
		1	2	3	4	5	6	7	
American Kestrel^c^	2	1.2 (1.0–1.3)	1.9 (1.6–2.1)	3.9 (3.0–4.2)	4.1 (2.8–4.4)	4.9 (4.6–5.1)	5.1 (4.6–5.3)	3.3 (3.1–3.4)	2 (100)
Northern Bobwhite	3	<–0.2	<–0.2	0.2 (<0.4–0.3)	<–0.2	<–0.2	<–0.2	<–0.2	1 (33)
Japanese Quail	3	<–0.2	<–0.2	<–0.2	<–0.2	<–0.2	2.5 (<0.4–3.0)	1.9 (<0.4–2.4)	1 (33)
Killdeer	2	NT	<0.1	0.4 (<0.4–0.7)	0.1 (<0.4–0.4)	0.8 (<0.4–1.1)	0.1 (<0.4–0.4)	0.4 (<0.4–0.7)	1 (50)
Ring-billed Gull^d^	2	NT	3.1 (2.0–3.3)	2.8 (1.8–3.0)	3.3 (2.6–3.6)	3.5	3.5	3.4	2 (100)
Mourning Dove	3	NT	<–0.2	0.8 (<0.4–1.2)	0.5 (<0.4–0.8)	0.5 (<0.4–0.8)	<–0.2	<–0.2	2 (67)
Monk Parakeet	3	<–0.2	<–0.2	<–0.2	<–0.2	<–0.2	<–0.2	<–0.2	0 (0)
Budgerigar	3	NT	<–0.2	<–0.2	<–0.2	<–0.2	<–0.2	<–0.2	0 (0)
Great Horned Owl	1	<0.4	1.3	3.1	<0.4	5.8	4.9	2.8	1 (100)
Northern Flicker	1	<0.4	<0.4	<0.4	<0.4	<0.4	<0.4	<0.4	0 (0)
Blue Jay^e^	2	0.9 (<0.4–1.2)	4.0 (1.4–4.3)	3.9 (2.5–4.2)	3.6	3.7	2.2	1.2	2 (100)
Black-billed Magpie	3	<–0.2	0.8 (<0.4–1.0)	2.1 (1.4–2.5)	3.1 (2.5–3.3)	4.0 (<0.4–4.4)	–	–	3 (100)
American Crow	6	<–0.5	2.3 (<0.4–2.5)	5.0 (1.6–5.5)	4.3 (3.1–4.7)	5.7	–	–	6 (100)
Fish Crow^f^	8	<–0.6	1.0 (<0.4–1.7)	3.4 (1.3–4.2)	3.6 (<0.4–4.1)	3.8 (1.8–4.4)	3.8 (1.6–4.6)	3.5 (1.9–4.1)	8 (100)

^a^Presented as mean log_10_ PFU/swab, with ranges. ^b^NT, not tested; –, no birds survived the infection to be sampled. ^c^American Kestrels were tested at 9, 10,and 11 days postinoculation, with the following results for days 9 and10, respectively: 2.3 (1.8–2.5); 1.8 (<0.4–2.1). WNV was not isolated from oral swabs collected on day 11. ^d^This Ring-billed Gull developed signs of illness after 7 days postinoculation and was euthanized at 13 days postinoculation, at which time an oral swab contained 10 PFU WNV. ^e^This Blue Jay was sampled at 8 and 9 days postinoculation. No virus was isolated from oral swabs. ^f^Surviving Fish Crows were tested at 8 and 9 days postinoculation with the following results for days 8 and 9, respectively: 3.6 (2.0–4.3); 2.1 (<–0.5–2.5).

### Viral Load and Viral Persistence in Organs

Some birds that died acutely were necropsied to determine viral load in different organs (Table 8). Almost all organs evaluated were infected, although certain organs harbored consistently more virus. Among the four species of corvids evaluated, titers were higher in American Crows and Blue Jays than in Fish Crows and Black-billed Magpies. Titers were lowest in Ring-billed Gulls, but most tissues were still infected.

**Table 8:** Viral load, determined by Vero plaque assay, in organs harvested from fatal cases of West Nile virus infection in experimentally infected birds^a^

Species	ID no.	Sex	Mode of infection	Organ (log_10_ PFU/0.5 cm^3^)										
				Br	Ki	He	Sp	Li	Lu	In	Es	Go	Sk	Ey
Ring-billed Gull	BDG	Male	M	2.7	2.8	1.0	3.3	3.0	3.2	<1.0	2.7	1.6	3.0	2.0
Ring-billed Gull	LG	Female	C	3.0	2.4	1.7	1.3	<1.0	<1.0	<1.0	2.3	3.0	2.4	3.0
American Crow	21	Male	C	7.2	7.6	5.4	7.9	7.5	8.5	7.6	7.8	6.8	4.4	7.7
American Crow	24	Female	M	6.0	8.2	7.7	7.0	7.3	7.1	8.4	7.4	7.0	4.6	6.6
American Crow	25	Female	M	5.9	7.2	5.7	6.7	6.8	6.7	10.6	6.2	7.9	3.8	6.9
American Crow	34	Female	C	8.3	8.2	7.8	7.9	7.7	8.3	9.0	8.2	8.1	5.3	7.9
American Crow	37	NR	C	7.2	8.2	7.7	7.8	7.4	8.5	10.0	7.3	NT	5.3	7.7
American Crow	41	Female	M	8.3	9.1	8.5	8.7	8.7	10.3	6.6	8.8	9.4	5.6	8.5
American Crow	42	Female	M	8.1	9.1	8.4	7.8	8.1	8.4	10.3	8.6	8.3	5.8	7.9
American Crow	529	Female	M	6.1	7.2	7.0	6.5	5.3	7.7	5.7	5.9	6.9	6.8	5.7
American Crow	543	Female	M	8.3	8.4	8.4	7.2	7.4	9.6	8.7	8.0	8.7	5.9	8.6
American Crow	562	NR	M	8.3	8.2	6.9	7.7	7.7	8.1	10.2	7.4	8.1	5.8	7.3
American Crow	574	Male	C	8.3	8.1	8.2	8.0	8.6	8.1	9.7	6.3	7.8	6.1	7.5
American Crow	805	Male	M	8.1	6.5	7.3	5.9	6.3	9.2	8.3	8.0	6.3	6.4	8.7
Fish Crow	005	Male	M	6.9	4.0	<2.0	3.3	3.5	4.0	4.5	8.5	4.4	3.0	6.2
Fish Crow	016	Male	M	2.7	3.2	6.6	1.0	1.0	4.6	5.2	5.1	3.6	4.0	4.8
Fish Crow	036	NR	M	3.0	3.1	6.6	3.3	1.5	5.3	3.0	6.5	3.3	4.9	3.1
Fish Crow	038	Male	M	4.1	3.9	4.7	1.3	1.0	4.4	2.0	5.4	4.5	2.8	5.0
Fish Crow	050	Female	M	6.9	8.1	8.5	8.2	7.8	7.6	8.6	7.7	8.0	5.5	6.9
Fish Crow	058	Male	M	4.5	5.1	6.9	2.4	1.6	5.8	6.0	5.1	2.7	5.0	5.3
Fish Crow	402	Male	U	4.7	3.6	3.8	3.7	1.5	3.0	2.8	7.4	1.8	3.7	5.8
Blue Jay	124	NR	M	7.4^b^	7.8	9.0	8.3	8.8	9.1	6.3	NT	7.1	NT	NT
Blue Jay	125	NR	M	7.3^b^	8.6	9.1	8.3	8.9	9.2	7.3	NT	7.3	NT	NT
Blue Jay	908	Male	C	8.2	9.0	8.8	8.6	8.5	9.0	8.0	8.7	9.1	6.9	7.9
Blue Jay	909	Male	C	2.7	6.1	6.6	5.5	4.3	5.8	3.4	4.5	5.0	5.9	7.1
Blue Jay	910	Female	M	8.2	9.0	9.0	8.1	8.4	9.1	7.9	8.9	9.1	6.0	8.0
Black-billed Magpie	LG	Male	M	4.7	6.6	6.6	3.9	5.4	6.5	4.8	6.7	5.9	5.5	6.3
Black-billed Magpie	RBLG	Male	M	5.9	5.5	5.0	4.0	4.8	4.4	2.7	5.9	4.8	4.1	4.6
Black-billed Magpie	NB	Male	C	5.8	6.2	7.2	5.9	6.4	5.7	5.1	5.9	6.5	4.8	5.5
Black-billed Magpie	RB	Male	C	5.0	7.7	6.2	4.5	6.8	6.3	5.2	5.8	4.7	5.9	5.3
Black-billed Magpie	RG	Female	M	6.4	5.4	6.7	4.7	1.9	6.1	1.0	6.6	4.5	5.9	4.7
Common Grackle	120	Male	M	3.5	>3.6	>3.6	>3.6	>3.6	>3.6	<1.0	5.0	<1.0	4.0	5.0
Common Grackle	123	Male	M	2.4	>3.3	NT	NT	NT	NT	<1.0	4.9	5.1	4.6	4.9
House Finch	0	Female	M	3.9	3.8	5.9	3.6	3.8	3.9	<1.0	5.5	3.8	4.1	6.2
House Finch	1	Male	M	4.9	3.0	6.1	3.5	2.7	6.0	<1.0	5.7	3.2	5.8	6.3

^a^BR, brain; Ki, kidney; He, heart; Sp, spleen; Li, liver; Lu, lung; In, intestine; Es, esophagus; Go, gonad; Sk, skin; Ey, eye; M, mosquito-exposed; C, contact-exposed; U, undetermined mode of transmission; NR, not recorded; and NT, not tested. ^b^Blue Jay brains were evaluated for viral load in both cerebellum and cortex. In both cases, cerebellum was negative.

All surviving birds were necropsied after euthanization to determine whether infectious WNV could be detected in any of 11 organs, including brain, eye, kidney, heart, spleen, liver, lung, intestines, gonads, esophagus, and skin. This analysis determined that 18 of 41 birds sampled at 14 dpi sustained virus infections in one or more organs for up to 13 days beyond the period of viremia and, in two cases, in birds with no detectable viremia (Table 9).

**Table 9:** Viral load, determined by Vero plaque assay in organs harvested from surviving birds 14 days after West Nile virus (WNV) infection by mosquito bite^a,b^

Species	ID no.	Sex	Organ (PFU/0.5 cm^3^)											Days postviremia
			Br	Ki	He	Sp	Li	Lu	In	Es	Go	Sk	Ey	
American Kestrel^c^	F2	F	–^d^	20	–	20	–	–	–	–	NT	30	–	10
American Kestrel	F3	F	–	–	–	10	–	–	–	–	NT	–	–	11
Japanese Quail	902	U	10	10	–	–	–	–	–	–	–	–	–	8
Japanese Quail	904	U	–	–	–	10	–	–	–	–	10	–	–	14
Japanese Quail	907	U	–	–	20	20	–	–	–	–	–	–	–	14
Killdeer	CT	U	–	60	–	–	–	–	20	–	–	110	–	9
Killdeer	WT	U	–	–	–	550	–	–	–	–	–	2x10^4^	–	10
Mourning Dove	LCW	U	–	20	–	–	–	–	–	–	–	–	–	10
Mourning Dove	RB	M	–	100	–	–	–	–	–	–	–	–	–	11
Budgerigar	13591	U	–	–	130	–	–	–	–	–	–	–	–	13
Blue Jay	911	U	20	–	–	–	–	–	–	–	–	–	360	9
Fish Crow	049	U	–	–	–	–	–	–	–	–	–	–	30	9
Red-winged Blackbird	711	M	–	–	10	–	10	–	–	–	–	–	–	11
Common Grackle	119	F	–	–	–	10	–	–	–	10	20	380	150	11
Common Grackle	122	M	–	–	–	–	–	–	–	–	–	–	10	10
House Sparrow	011	F	10	50	–	–	–	–	40	90	–	370	60	8
House Sparrow	012	M	–	–	–	120	–	590	10	–	–	10	–	10
House Sparrow	016	M	200	20	–	50	–	20	–	–	–	–	50	8

^a^For each bird, the number of days postviremia is indicated. Birds from which no virus was detected are not included. WNV was isolated from at least one organ sample from 18 birds, and at least one isolate was made from each of the 11 different organs. Liver had the fewest isolates with one; spleen had the most with eight. Titers were generally low. The highest titered specimen was a skin sample from a Killdeer. Twenty-three surviving birds had no WNV isolated from tissues at 14 days postinoculation, including three Northern Bobwhite, three Ring-necked Pheasants, three Monk Parakeets, two Budgerigars, one Great Horned Owl, one Mourning Dove, six European Starlings, two Common Grackles, and two Red-winged Blackbirds. In addition, five Rock Doves were sampled at 64 days postinoculation and were negative. ^b^M, male; F, female; U, undetermined gender or gender not recorded; Br, brain; Ki, kidney; He, heart; Sp, spleen; Li, liver; Lu, lung; In, intestine; Es, esophagus; Go, gonad; Sk, skin; Ey, eye; and NT, not tested. ^c^Kestrels were tested 15 days postinoculation. ^d^ – indicates that no virus was isolated (threshold of detection 10 PFU/0.5 cm^3^ tissue).

### Reservoir Competence

We analyzed viremia data from mosquito-exposed birds to determine values for susceptibility, mean infectiousness, and duration of infectious viremia; from these, we calculated competence indices (Table 10). Species with high mean peak viremias and long duration of viremia generally also had high competence index values.

**Table 10:** West Nile virus reservoir competence index values derived for 25 species of birds

Common name	Susceptibility	Mean infectiousness	Mean duration (days)	Reservoir competence index
Blue Jay	1.0	0.68	3.75	2.55
Common Grackle	1.0	0.68	3	2.04
House Finch	1.0	0.32	5.5	1.76
American Crow	1.0	0.5	3.25	1.62
House Sparrow	1.0	0.53	3	1.59
Ring-billed Gull	1.0	0.28	4.5	1.26
Black-billed Magpie	1.0	0.36	3	1.08
American Robin	1.0	0.36	3	1.08
Red-winged Blackbird	1.0	0.33	3	0.99
American Kestrel	1.0	0.31	3	0.93
Great Horned Owl	1.0	0.22	4	0.88
Killdeer	1.0	0.29	3	0.87
Fish Crow	1.0	0.26	2.8	0.73
Mallard	1.0	0.16	3	0.48
European Starling	1.0	0.12	1.8	0.22
Mourning Dove	1.0	0.11	1.7	0.19
Northern Flicker	1.0	0.06	1	0.06
Canada Goose	1.0	0.1	0.3	0.03
Rock Dove	1.0	0	0	0
American Coot	1.0	0	0	0
Japanese Quail	1.0	0	0	0
Northern Bobwhite	1.0	0	0	0
Ring-necked Pheasant	1.0	0	0	0
Monk Parakeet	1.0	0	0	0
Budgerigar	0.7	0	0	0

## Discussion

### Reservoir Competence

The principal goal of our experimental infection studies was to estimate reservoir competence for a variety of candidate reservoir species in the United States. We used a formula derived for evaluating vertebrate reservoir competence for *Eastern equine encephalomyelitis virus* (family: *Togaviridae*), a mosquito-borne alphavirus (*12*). The value derived for reservoir competence is an index that reflects the relative number of infectious mosquitoes that would be derived from feeding on these hosts. This value depends on the concentration of infectious virus particles in blood and the duration of an infectious level viremia. We have shown that WNV viremia profiles derived from mosquito-borne infection in birds vary greatly among the 25 species that we evaluated. Birds that sustained a viremic titer greater than 10^5.0^ PFU/mL were considered infectious for *C. pipiens* (*13*) and *C. quinquefasciatus* (*14*), two important enzootic vectors. These bird species were considered reservoir competent for WNV, whereas species that did not develop viremia of sufficient titer to infect these mosquitoes were considered incompetent. Some mosquito vectors may develop infections after imbibing lesser concentrations of virus. For example, the threshold WNV titer for infection of *C. univittatus* in South Africa was reported as <10^4.0^ 50% suckling mice lethal doses (SMLD_50_)/mL blood, and <10^4.6^ SMLD_50_/mL blood for infection of *C. perexiguus* in Egypt (*15*). Recent studies with WNV (New York 1999) indicate that some California populations of *C. tarsalis*, *C. pipiens*, and *C. erythrothorax* are susceptible to infection at titers <10^5.0^ PFU/mL (*16*). Similarly, the threshold for SLEV infection of *C. tarsalis* may be as low as 10 PFU per bloodmeal (approximately 10^3.3^ PFU/mL blood) (*17*). Nonetheless, titers <10^5.0^ PFU/mL probably result in few mosquito infections.

Our observation that passerine bird species were generally competent for WNV transmission is consistent with the role of these birds as hosts for other flaviviruses such as SLEV and Japanese encephalitis virus (*18*,*19*). Previous work with African strains of WNV also implicated passerine birds as the most competent. In Egypt, experimental infections of House Sparrows and Hooded Crows (*Corvus corone sardonius*), both passerine species, using a local strain of WNV (Ar-248), showed that these two species developed high titered viremia whereas three nonpasserine species (including a dove, an egret, and a falcon) were weakly competent (*20*). Adult chickens and pigeons were incompetent (*21*). Similarly, in South Africa, inoculation of 14 bird species with a local WNV strain showed two highly competent species, both passerine (Masked Weaver [*Ploceus velatus*] and Red Bishop [*Euplectes orix*], both closely related to House Sparrow) (*22*). Our finding that Anseriformes were weakly competent reservoir hosts for WNV is consistent with the findings of the South African study, which evaluated three species of ducks. Our study also coincided with both the Egyptian and South African studies in finding Rock Doves (pigeons) incompetent but other species of doves weakly competent. Reservoir competence index values derived from experimental infection studies must be combined with field-derived data to fully evaluate the importance of candidate reservoir hosts in a specific location. However, larger sample sizes should be studied to derive competence values with greater accuracy.

### Illness and Death Associated with WNV infection

Our study confirms that some species of bird suffer high mortality rates from WNV infection. The mortality rates presented in this paper are preliminary because of small sample sizes, inadequate controls, and bias from the effects of captivity and handling. Nonetheless, birds sufficiently weakened by the infection to succumb in captivity are also likely to succumb in nature, where other stresses may contribute to death. Avian deaths were not reported in natural WNV infections until 1998 when domestic goslings in Israel were affected, as well as White storks (*Ciconia ciconia*) (*23*). The 1998 goose strain is essentially identical to the New York 1999 strain that resulted in thousands of bird deaths beginning in 1999 in New York City (*24*).

The high mortality rate in corvids was presaged by the results of the Egyptian experimental infection study, in which all 13 infected Hooded Crows succumbed (*20*). However, the lack of observed crow deaths and the observation of high seroprevalence in natural crow populations (an indicator of survival of infection) led investigators in Egypt to speculate that the crow deaths in captivity were artifacts (*21*).

The observed high mortality rate in 8 of the 25 species tested in our study indicates that these 8 species (Table 4) may be useful in avian mortality surveillance. These species include all the corvids tested, as well as House Sparrow and Common Grackle, two abundant passerine bird species likely to be important reservoir hosts in some locations, and Ring-billed Gull. Deaths in experimentally infected passerines (House Sparrows and crows) have been reported previously (*21*). The Egyptian and the South African studies did not include Charadriiformes. However, in a Russian study of WNV infection in Black-tailed Gulls (*Larus crassirostris*), deaths were observed (*25*), as well as in naturally infected White-eyed Gulls (*Larus leucophthalmus*) in Israel (*23*).

The lack of clinical signs and death in 17 species suggests that mortality rates in these birds are low. However, natural WNV-associated deaths have been reported from all 10 orders of birds included in this study (*11*). Even for birds that are generally resistant to fatal WNV infections, the virus may still be an important cause of death relative to the overall mortality rate of the population; this idea provides an explanation for why 7 (17%) of 41 dead pigeons tested positive for WNV in New York in 2000 (*11*) and yet none of 6 pigeons experimentally infected showed signs of illness. Whether WNV alone is capable of killing a pigeon is unknown; WNV infection may require underlying illness or immune suppression in a pigeon to result in death.

### Oral Transmission

We have demonstrated that certain bird species may become infected by WNV (New York 1999) after ingesting it in infected dead animals and infected mosquitoes, both natural food items of some species. This phenomenon was previously observed in American Crows that ingested WNV-infected suckling mice (*26*) and in mammals on several occasions (*27*). Langevin et al. (*28*) were not able to infect chickens orally. We found that the viremia profiles generated by oral infection were essentially identical to those derived from mosquito-borne infection, although in some species, onset of viremia was delayed by approximately 1 day. The importance of oral WNV infection in birds is unknown but may contribute to the success of avian mortality surveillance compared with surveillance for infected mosquitoes (*5*,*29*) or other surveillance systems. Numerous dead crows may result from a single mosquito-borne transmission to a bird or mammal because of the carrion-feeding behavior of crows.

### Contact Transmission

We have shown that certain bird species may become infected by WNV (New York 1999) after being in close contact with other infected birds, in the absence of mosquito-borne transmission. We observed this phenomenon in American Crows, Blue Jays, Black-billed Magpies, and Ring-billed Gulls. This type of direct transmission of WNV among birds was first reported in a cage mate of a chicken inoculated by needle (*28*). Subsequently, this transmission was observed in needle-inoculated domestic goslings (*30*) and American Crows held in a free-flight aviary with uninfected crows (*26*). The mode of this “cage mate transmission” is unknown. Viremia profiles in contact-exposed American Crows, Blue Jays, and Black-billed Magpies were indistinguishable from those of mosquito-exposed birds. Onset of viremia in contact-exposed crows and magpies occurred approximately 1 day after death of the mosquito-exposed cage mate, suggesting that exposure did not occur until death of the infected companion. Onset of viremia in contact-exposed Blue Jays, however, began before the death of the mosquito-exposed cage mates. Whether direct transmission of WNV between birds occurs in nature in the absence of mosquitoes is unknown. However, given the close cloacal and oral contact between birds that occurs within families during the breeding season and the sometimes high quantity of WNV in oral and cloacal fluids, this type of transmission likely occurs in nature.

### Viral Shedding

In general, arboviruses are not thought to be shed by their hosts because of the requirement for arthropod vectors in the transmission cycle. However, Langevin et al. (*28*) detected low-level shedding in WNV-infected chickens by swabbing the cloaca and the oral cavity. We extended those observations to many other species of birds in this study. Viral shedding may be involved in the cage mate transmission that we observed in corvids and Ring-billed Gull. The prospect of shedding in naturally infected birds has other implications for both public health and potential spread of WNV within and between species. Does shedding in birds represent a health risk to humans? Can birds shed virus to the extent that other birds in close contact can become infected in the absence of mosquitoes? These questions require further study. Our observations of shedding in acutely infected birds led to the hypothesis that swabbing corvid carcasses could be useful for diagnosing WNV infection. This hypothesis was validated (*31*).

### Viral Persistence and Viral Load in Organs

Several reports have suggested that WNV and other flaviviruses may persist in the organs of birds in such a way as to permit retransmission to vector mosquitoes after the period of initial viremia (*32*–*34*). The mode of retransmission, however, is unknown. To generate preliminary data on persistence of the New York 1999 strain of WNV in North American bird species, we monitored persistence of virus infection in organs of 42 surviving birds that were euthanized at 14 dpi. Almost half of these birds harbored infectious virus in organs. Although we isolated WNV in all 11 organs evaluated, we found no pattern of which organs are infected for which species, and viral titers were generally low. Organs most frequently infected were spleen, kidney, skin, and eye; the liver was the least likely to harbor infectious virus particles. These infections may have been remnants of the acute infection. However, some unexpected observations were made in these studies. For example, the organ with the highest density of infectious virus particles was a skin sample taken from a Killdeer. Skin samples were positive in six birds tested, including an American Kestrel, both Killdeer, a Common Grackle, and two House Sparrows (and high titers were consistently detected in skin samples collected from fatal infections). Persistent skin infections may be transmitted to mosquitoes that contact the skin while feeding even beyond the period of infectious-level viremia. Infected skin also suggests that ticks may become infected with WNV while feeding for several days within the skin. Interestingly, charadriiform birds are thought to serve as reservoir hosts in a soft tick-borne WNV transmission cycle in Eurasia (*35*). Another unexpected finding was infection of ovaries; persistent infections in these organs raise the possibility of transovarial transmission.

We also evaluated the viral load of organ samples from seven species of birds that died from WNV infection. Overall, our evaluation of viral loads in 11 organs of WNV-infected carcasses supports the current prioritization of brain or kidney for selective organ testing for WNV (*36*–*38*). Intestines had the highest WNV concentrations of the organs evaluated, yet several birds (the two gulls and two finches) had no detectable infectious WNV in their intestines. Although skin titers were lower, the universal positivity among the birds tested and the ease of specimen collection in the field warrant consideration of skin as a potential biologic specimen for collection from carcasses in the field.

### Development of Neutralizing Antibodies

We expected all infected birds to generate a humoral immune response to WNV, with development of virus-neutralizing antibodies. Thus, the two Budgerigars that did not produce detectable antibodies (assayed at 14 dpi) were thought to have avoided infection. However, one of these parakeets harbored a persistent infection in heart tissue, indicating that infection did occur. This same bird had detectable viremia only at 1 dpi. With one species, Rock Doves, we followed the immune response through 9 weeks postinfection. All six Rock Doves generated a neutralizing immune response that persisted throughout the monitoring period. An early rise-fall-rise pattern in the neutralizing antibody response (Figure 3) is probably explained by the early, ephemeral contribution of immunoglobulin (Ig) M to virus neutralization, followed by a rise in neutralizing IgY. The Rock Dove (or domestic pigeon) is considered a candidate sentinel for monitoring WNV transmission in the United States (*29*). Our results indicate that Rock Doves have a strong immune response after a brief, low-level (noninfectious) viremia, both important criteria for candidate sentinels.

## Conclusion

We have presented basic data on the course of WNV (New York 1999) infection in 25 species of birds, including viremia duration and magnitude, illness and death, persistent infection in organs, and viral shedding. We have also shown that some birds are susceptible to oral transmission and that some cage mates may become infected in the absence of mosquitoes, although the mode of this type of transmission remains unknown. An analysis of our data shows that passerine birds, charadriiform birds, and at least two species of raptors (American Kestrel and Great Horned Owl) are more competent than species evaluated from the following orders: Anseriformes, Columbiformes, Galliformes, Gruiformes, Piciformes and Psittaciformes. Indeed, many birds of the latter orders were found to be incompetent for transmission.

## Supplementary Material

Appendix A Plaque-Reduction Neutralization Assay

Appendix B Venipuncture

Appendix C Plaque Assay

Appendix D Standard curve lookup table relating viremia titer (log10 PFU/mL) to infectiousness for Culex pipiens
