# Supplementary material for: Experimental Infection of North American Birds with the New York 1999 Strain of West Nile Virus
Source: Emerg Infect Dis. 2003 Mar;9(3):311–22. doi: 10.3201/eid0903.020628 (PMC2958552; doi:10.3201/eid0903.020628)
Supplement: Appendix A — Plaque-Reduction Neutralization Assay [file 02-0628_appA-s1.pdf]

Plaque-Reduction Neutralization Assay

Serum samples were assayed for West Nile virus (WNV)–specific antibodies by using the plaque-reduction neutralization test as previously described (*1*). Briefly, each serum sample was diluted 1:5 in BA1 and mixed with an equal volume of BA1 containing a suspension of WNV NY99-4132 at a concentration of approximately 200 PFU/0.1 mL, such that the final serum dilution was 1:10 and the final concentration of WNV was approximately 100 PFU/0.1 mL. For the postinoculation serum samples, we also tested serial twofold dilutions of serum to determine endpoint 90%-neutralization titers. In most cases, preinoculation serum samples were tested for neutralizing antibodies to St. Louis encephalitis virus, a closely related flavivirus that may cross-react serologically with WNV (*2*) and may partially protect against WNV infection (*3*). After 1 h incubation at 37°C, 5% CO_2, virus-serum mixtures were assayed for virus content by plaque assay (see above). Controls used included BA1 only (cell viability control), bird serum-free virus mixture with BA1 only (to enumerate the number of PFU in the challenge dose of virus), and WNV hyperimmune mouse ascitic fluid (diluted 1:200) mixture with virus (positive control, to verify challenge virus identity).

Appendix A References

1. Beaty BJ, Calisher CH, Shope RE. Arboviruses. In: Schmidt NJ, Emmons RW, editors. Diagnostic procedures for viral, rickettsial and chlamydial infections, 6th ed. Washington: American Public Health Association; 1989. p. 797–855.
2. Calisher CH, Karabatsos N, Dalrymple JM, Shope RE, Porterfield JS, Westaway EG, et al. Antigenic relationships between flaviviruses as determined by cross-neutralization tests with polyclonal antisera. J Gen Virol 1989;70:37–43.
3. Tesh RB, Travassos da Rosa AP, Guzman H, Araujo TP, Xiao SY. Immunization with heterologous flaviviruses protective against fatal West Nile encephalitis. Emerg Infect Dis 2002;8:245–51.
