# Supplementary material for: Experimental Infection of North American Birds with the New York 1999 Strain of West Nile Virus
Source: Emerg Infect Dis. 2003 Mar;9(3):311–22. doi: 10.3201/eid0903.020628 (PMC2958552; doi:10.3201/eid0903.020628)
Supplement: Appendix B — Venipuncture [file 02-0628_appB-s2.pdf]

Venipuncture

We collected blood samples from each bird before infection to confirm seronegative status for West Nile virus (WNV). Additional samples were collected once a day for 7 days postinoculation (dpi) at 24-h intervals, and a final sample was collected 1 week later to confirm seroconversion. We collected blood with 1-mL syringes with attached sub-Q needles (27-gauge needles for birds $\leq$60 g in mass or 26-gauge for all other birds.) Initial and final blood samples ranged in volume from 0.2–0.6 mL, depending on the size of the bird, and were collected in Microtainer serum separator tubes (Becton, Dickinson and Co., Franklin Lakes, NJ). Specimens were held at ambient temperature for at least 30 min and then centrifuged for 3 min at 7,500 rpm in a refrigerated Eppendorf centrifuge (Model 5417R; Brinkmann Instruments, Inc., Westbury, NY). Once a day, blood samples (0.1 mL for birds $\leq$60 g in mass or 0.2 mL for all other birds) were diluted in 4.5 V of BA1 diluent (composed of Hank's M-199 salts, 1% bovine serum albumin, 350 mg/L sodium bicarbonate, 100 U/mL penicillin, 100 mg/L streptomycin, and 1 mg/L Fungizone in 0.05 M Tris, pH 7.6) in a 2-mL cryovial. After approximately 30 min at ambient temperature (to permit coagulation), these cryovials were placed temporarily on wet ice and then centrifuged cold at 3,750 rpm for 10 min in a refrigerated Beckman centrifuge (Model GS-6R, Beckman Instruments, Inc., Palo Alto, CA), effectively separating the clot from a 1:10 dilution of serum. Centrifuged cryovials were then stored at –70°C until titrated by Vero plaque assay.
