# Supplementary material for: Experimental Infection of North American Birds with the New York 1999 Strain of West Nile Virus
Source: Emerg Infect Dis. 2003 Mar;9(3):311–22. doi: 10.3201/eid0903.020628 (PMC2958552; doi:10.3201/eid0903.020628)
Supplement: Appendix C — Plaque Assay [file 02-0628_appC-s3.pdf]

Plaque Assay

Plaque assay for detecting and titrating infectious WNV particles in blood samples, cloacal and oral swabs, and organ tissues was carried out by using a double overlay system in Vero (a continuous line of African Green Monkey kidney cells) culture. Most blood specimens were titrated after a single freeze-thaw cycle. Organ samples were titrated after one or two freeze-thaw cycles. Some high-titered specimens were titrated by using serial 10-fold dilutions in BA1 after an additional freeze-thaw. After inoculating cell monolayers with 0.1 mL (in duplicate) and incubating the sample for 1 h at 37°C, 5% CO_2, each well was overlaid with 3 mL of 0.5% agarose in M-199 medium supplemented with 350 mg/L sodium bicarbonate, 29.2 mg/L L-glutamine, and antibiotics as in BA1. After 48 h of additional incubation, a second 3-mL 0.5% agarose overlay containing 0.004 % neutral red dye was added for plaque visualization. Plaques were counted on days 3 and 4 after infection of the Vero cells.
