# Supplementary material for: Experimental Infection of North American Birds with the New York 1999 Strain of West Nile Virus
Source: Emerg Infect Dis. 2003 Mar;9(3):311–22. doi: 10.3201/eid0903.020628 (PMC2958552; doi:10.3201/eid0903.020628)
Supplement: Appendix D — Standard curve lookup table relating viremia titer (log10 PFU/mL) to infectiousness for Culex pipiens [file 02-0628_appD-s4.pdf]

Appendix D Table. Standard curve lookup table relating viremia titer ($\log_{10}$ PFU/mL) to infectiousness for *Culex pipiens*

| Viremia | Infectiousness | Viremia | Infectiousness | Viremia | Infectiousness | Viremia | Infectiousness |
|---|---|---|---|---|---|---|---|
| 5.0 | 0.02 | 7.0 | 0.22 | 9.0 | 0.42 | 11.0 | 0.62 |
| 5.1 | 0.03 | 7.1 | 0.23 | 9.1 | 0.43 | 11.1 | 0.63 |
| 5.2 | 0.04 | 7.2 | 0.24 | 9.2 | 0.44 | 11.2 | 0.64 |
| 5.3 | 0.05 | 7.3 | 0.25 | 9.3 | 0.45 | 11.3 | 0.65 |
| 5.4 | 0.06 | 7.4 | 0.26 | 9.4 | 0.46 | 11.4 | 0.66 |
| 5.5 | 0.07 | 7.5 | 0.27 | 9.5 | 0.47 | 11.5 | 0.67 |
| 5.6 | 0.08 | 7.6 | 0.28 | 9.6 | 0.48 | 11.6 | 0.68 |
| 5.7 | 0.09 | 7.7 | 0.29 | 9.7 | 0.49 | 11.7 | 0.69 |
| 5.8 | 0.10 | 7.8 | 0.30 | 9.8 | 0.50 | 11.8 | 0.70 |
| 5.9 | 0.11 | 7.9 | 0.31 | 9.9 | 0.51 | 11.9 | 0.71 |
| 6.0 | 0.12 | 8.0 | 0.32 | 10.0 | 0.52 | 12.0 | 0.72 |
| 6.1 | 0.13 | 8.1 | 0.33 | 10.1 | 0.53 | | |
| 6.2 | 0.14 | 8.2 | 0.34 | 10.2 | 0.54 | | |
| 6.3 | 0.15 | 8.3 | 0.35 | 10.3 | 0.55 | | |
| 6.4 | 0.16 | 8.4 | 0.36 | 10.4 | 0.56 | | |
| 6.5 | 0.17 | 8.5 | 0.37 | 10.5 | 0.57 | | |
| 6.6 | 0.18 | 8.6 | 0.38 | 10.6 | 0.58 | | |
| 6.7 | 0.19 | 8.7 | 0.39 | 10.7 | 0.59 | | |
| 6.8 | 0.20 | 8.8 | 0.40 | 10.8 | 0.60 | | |
| 6.9 | 0.21 | 8.9 | 0.41 | 10.9 | 0.61 | | |
